# Fish Scale Valorization by Hydrothermal Pretreatment Followed by Enzymatic Hydrolysis for Gelatin Hydrolysate Production

**DOI:** 10.3390/molecules24162998

**Published:** 2019-08-19

**Authors:** Yiqi Zhang, Dan Tu, Qing Shen, Zhiyuan Dai

**Affiliations:** 1 Institute of Seafood, Zhejiang Gongshang University, Hangzhou 310035, China; 2 Key Laboratory of Aquatic Products Processing of Zhejiang Province, Hangzhou 310035, China

**Keywords:** gelatin hydrolysate, fish scale, hydrothermal, molecular weight distribution, ACE inhibitory

## Abstract

Protein hydrolysates from fish by-products have good process suitability and bioavailability in the food industry. The objective of this work was to develop a method for protein recovery from fish scales and evaluate the hydrolysis of the scale protein. The effect of the hydrothermal process on protein recovery, degree of hydrolysis (DH) and structural properties of the hydrolysates was investigated. Results showed that hydrothermal treatment could enhance protein recovery of tilapia scales without demineralization and dramatically improve the DH of the hydrolysates. The hydrothermal treated scales showed a better protein recovery (84.81%) and DH (12.88%) and released peptides more efficiently than that of the conventional treated samples. The obtained gelatin hydrolysates mainly distributed in the range of 200–2000 Da with an angiotensin I-converting enzyme (ACE) IC_50_ value of 0.73 mg/mL. The ACE inhibitory activity of gelatin hydrolysates was stable under high temperature, pH and gastrointestinal proteases. Hydrothermal treatment followed by enzymatic hydrolysis offers a potential solution for preparation of gelatin hydrolysates for food ingredients from fish processing by-products.

## 1. Introduction

Gelatin is widely used in biomedical, cosmetic, leather, food and pharmaceutical industries [1,2]. Fish gelatin has received increasing attention, because of the safety and religious concern of the mammalian analogue [3]. Correspondingly, fish processing by-products containing a large amount of collagen, such as fish skin, fin, bone and scale, are becoming potential alternative sources for gelatin production [4,5,6]. Although the nutritional value of gelatin is quite low for humans due to the lack of some essential amino acids, once it is hydrolyzed, gelatin hydrolysates have good process suitability and bioavailability, making them potential sources for value-added foods [7,8]. 

Gelatin hydrolysates from aquatic by-products are generally obtained by two separate operations. Firstly, gelatin is extracted from raw materials by a chemical pretreatment with dilute acid or alkali [1], which destabilizes the triple-helix through a disruption in hydrogen bonding and some covalent cross-links, thus enhancing protein solubilization. Gelatin hydrolysates are then produced by enzymatic hydrolysis of the extracted gelatin based on the commercial proteases’ specificity [1]. Nevertheless, chemical pretreatment is often harmful to the environment. In addition, the overall extraction efficiency of gelatin is typically low, limiting industrial output [9]. Taken together, the conventional methods used for the preparation of gelatin hydrolysates are generally reactant-, cost- and time-consuming and even bad for the environment. A simple and efficient extraction technology for gelatin hydrolysates is thus needed.

Recently, application of hydrothermal pretreatment in hydrolysis of macromolecule substance has been investigated. Under hydrothermal conditions, water has unique features, such as increased ionic products and a structural change in the hydrogen bond [10]. The increase in ionic product drives the formation of H_3_O^+^ and OH^−^ ions, which facilitates water to appear as an acid or base catalyst [11]. The autoclave batch reactor is one of the most widely used tools for hydrothermal processing because there is no need for a pumping system, in spite of the relatively long heating and cooling period time [12]. Recently, hydrothermal pretreatment has been successfully used to generate protein hydrolysates or free amino acids from different protein sources, including plant materials [13], fish by-products [11,14], animal by-products [15] and even pure protein like *β*-casein [10] and bovine serum albumin [16]. However, there is no detailed information on hydrothermal pretreatment for the extraction of gelatin from fish scales.

Tilapia is one of the major farmed species in China with a production over 1.62 million tons in 2018 [17]. Large quantities of fish scales are inevitably generated from fish fillet factories and are mostly disposed as solid wastes. Fish scale has plywood-like structures of dense layers of collagen (generally type I), covered with a mineral phase of calcium-deficient hydroxyapatite (HAP) [18]. These structure types render it resistant to most commercial proteases. As collagenous fiber and hydroxyapatite is linked tightly in the matrix of fish scale, a time- and reactant-consuming demineralization pretreatment is inevitable for collagen or gelatin extraction [19]. Although acidic and alkaline extraction have been developed for protein recovery from fish scales [20], hydrothermal pretreatment might be an alternative solution for improving the accessibility of fish scale to commercial proteases and enhancing the production of gelatin hydrolysates. 

The objective of this study was to develop a method for the recovery of protein from tilapia scale and evaluate the hydrolysis of scale protein by alcalase. In this work, the effects of hydrothermal processing on the protein recovery, degree of hydrolysis (DH), molecular mass distributions and angiotensin I-converting enzyme (ACE) inhibitory activity were investigated. The structural properties of the pretreated fish scale were further characterized by Fourier transform infrared (FTIR) spectroscopy. The results from this study could be beneficial to environment protection and utilization of by-products in fish processing industry.

## 2. Results and Discussion

### 2.1. Effect of Hydrothermal Pretreatment on Protein Recovery

**Figure 1:**
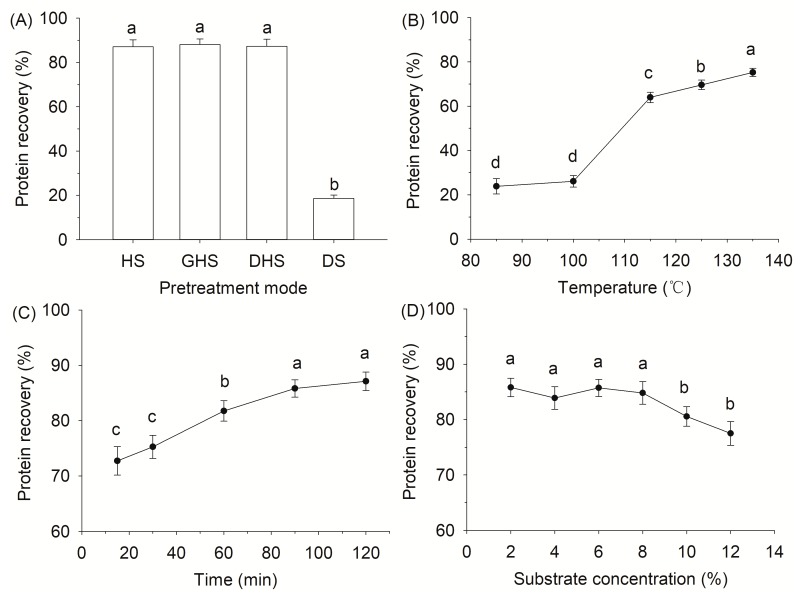
Effect of hydrothermal pretreatment on protein recovery of fish scales: (I) pretreatment mode (**A**), fish scales (HS), ground scales (GHS) and decalcified scales (DHS) after hydrothermal pretreatment (135 °C for 90 min) and decalcified scales (DS); (II) effect of temperature (**B**), time (**C**) and substrate concentration (**D**) on protein recovery of fish scales during the hydrothermal treatment. Different letters indicate that the results differ significantly (*p* < 0.05).

The tilapia scale possesses a chemical composition of 55.87% crude protein and 36.48% ash in the dry basis. The scale has similar amount of protein content compared with other fish scales, including the red drum fish [21] and spotted golden goatfish [22], indicating that the tilapia scale is a potential source for extracting collagen or gelatin. Different processing methods may have different impacts on the protein recovery of fish scales. In the present work, fish scales treated by the conventional method showed a protein recovery of 18.59% (Figure 1A). A similar yield (22.1%) was also found in gelatin extraction from Asian carp scales after the removal of Ca salt using 0.2 M EDTA [23]. As shown in Figure 1A, the protein recovery of hydrothermal treated fish scales was approximately 4.5 times higher than that of the conventional treated samples. The influence of a different pretreatment mode on the protein recovery of fish scales was further evaluated. However, no obvious differences (*p* > 0.05) in protein recovery were observed among the hydrothermal pretreatments with or without decalcification or pulverization. It indicates that the hydrothermal process is a promising technology for the recovery of fish scale protein free of catalysts or any other chemical resulting in secondary pollution. 

In this work, a simple hydrothermal process was developed for the extraction of protein from fish scales without decalcification or pulverization. The crude protein, ash and moisture contents of tilapia scale protein powder were 97.8%, 1.5% and 0.2%, respectively. The high protein content indicated that the recovered scale protein could be used to improve the nutrition or function properties of food products [14]. The hydrothermal treated fish scales showed higher protein recovery as compared to the conventional method. It could be speculated that the cross-linking between collagen and HAP might be decomposed during hydrothermal treatment, enhancing the extractability of fish scale protein. A recent study found that high temperature (135 °C) during the extrusion process could also help decompose the matrix of fish scale and enhance the separation and extraction of collagen [19]. Although the hydrothermal technique has been recently used to facilitate peptides or amino acids extraction from fish skin [11], porcine skin [12] and soy protein [13], to the best of our knowledge, this is the first report to accelerate the dissolubility of fish scale protein through hydrothermal process without demineralization.

Protein recovery efficiency mainly depends on the extraction condition, such as substrate concentration, pH, extraction temperature and time. Thus, the effect of operating parameters on protein recovery of fish scale was further studied. The effect of extraction temperature was evaluated at first. The protein recovery rate of fish scales was very low under mild thermal conditions and increased sharply (*p* < 0.05) from 23.82% to 75.27% with the increasing temperature (Figure 1B). A recent study from Olatunji and Denloye [24] found that the yield of collagen/gelatin extracted from croaker fish (*Pseudotolitus elongatus*) scales increased from 6% at 70 °C to 30% at 100 °C. Under hydrothermal condition, water is a strong source of H_3_O^+^ and OH^−^ ions [25], which could catalyze hydrolysis of collagen fibers into gelatin, peptides or amino acids through disruption of hydrogen bonding and some covalent cross-links [12]. The results indicated that hydrothermal treatment might be an alternative process to the conventional method based on acid or alkali extraction of gelatin from fish scales. The effect of pretreatment time on protein recovery at 135 °C was then investigated. The protein recovery reached a maximum at 90 min, after which there was no obvious difference (*p* > 0.05) (Figure 1C). To study the effect of the substrate concentration, the experiment was carried out under the condition of 135 °C for 90 min. As shown in Figure 1D, there was no obvious change in protein recovery (84.81%) with the substrate concentration raising from 2% to 8%. When the substrate concentration exceeded 8%, the protein recovery of fish scales decreased significantly (*p* < 0.05). The reason for the observations could be due to the increasing viscosity, causing mass and heat transfer problems [26]. Considering the results of protein recovery, conditions of a substrate concentration of 8% (*w*/*v*) at 135 °C for 90 min were selected for gelatin extraction from fish scales in this work.

### 2.2. FTIR

**Figure 2:**
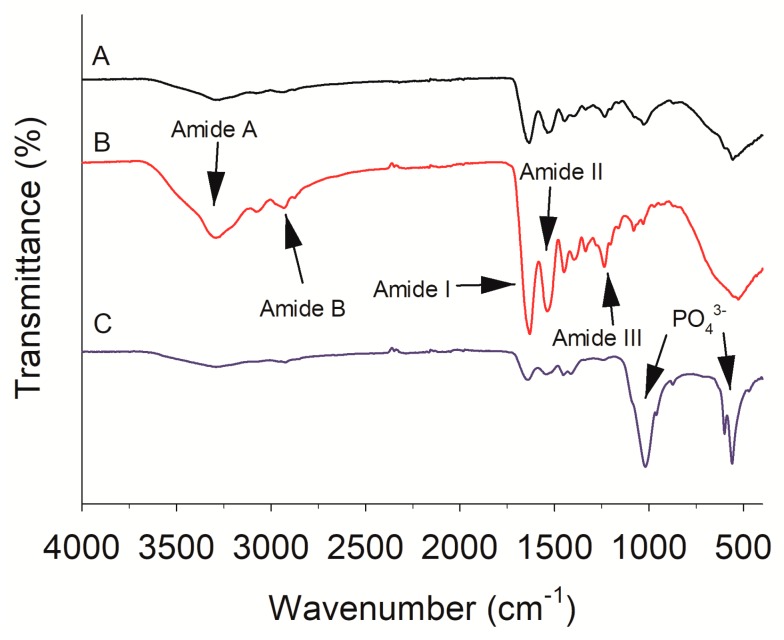
Fourier transform infrared spectra of fish scale under different pretreatment: untreated scales (**A**), tilapia scale protein powder (**B**), scale residues after hydrothermal treatment (**C**).

Studies revealed that FTIR spectra of protein contained several bands representing amide A (3304–3315 cm^−1^), amide B (2922–2940 cm^−1^) as well as amide I (1600–1700 cm^−1^), amide II (1550–1600 cm^−1^) and amide III (1220–1320 cm^−1^) [27]. In this work, FTIR spectra showed more prominent characteristic peaks of amide A, B and amide I, II and III in the extracted protein than the raw fish scale and residue (Figure 2), indicating that protein was extracted from the fish scales through the hydrothermal process.

The peaks at 3293 and 2934 cm^−1^ in amide A and B, was more pronounced in the extracted protein (Figure 2). The broadened peak at 3293 cm^−1^ in scale gelatin is mainly from the OH group, which could connect to other functional groups through hydrogen bonds [28]. The two peaks near 3078 and 2934 cm^−1^ are mainly from N-H and CH_2_ stretching vibrations. The amide I band appeared at 1631 cm^−1^ for the extracted protein is mainly due to stretching vibration of C=O. The amide II band, primarily associated with N-H bending and C–N stretching vibrations, was observed at 1537 cm^−1^. Amide III band (1220–1320 cm^−1^) is related to O=C–N and N-H bending and C–N stretching vibrations [29]. The presence of amide III band around 1236 cm^−1^ indicates that helical structure of the collagen/gelatin extracted [30]. The results showed that the tilapia scale gelatin extracted through hydrothermal process showed a secondary structure. On the other hand, the fish scale residue showed a strong absorption peak at about 1000–1100 cm^−1^ assigned to asymmetric stretching of phosphate group (PO_4_^3−^) [31], while the corresponding peaks of fish scale and the extracted collagen/gelatin were weaker (Figure 2). The more visible bands at 600 and 545 cm^−1^ indicate the characteristic peaks corresponding to PO_4_^3−^ groups of fish scale residue [32]. The above related peaks of HAP in fish scale residue suggests the removal of HAP from the extracted protein.

### 2.3. Effect of Hydrothermal Pretreatment on Enzymatic Hydrolysis

**Figure 3:**
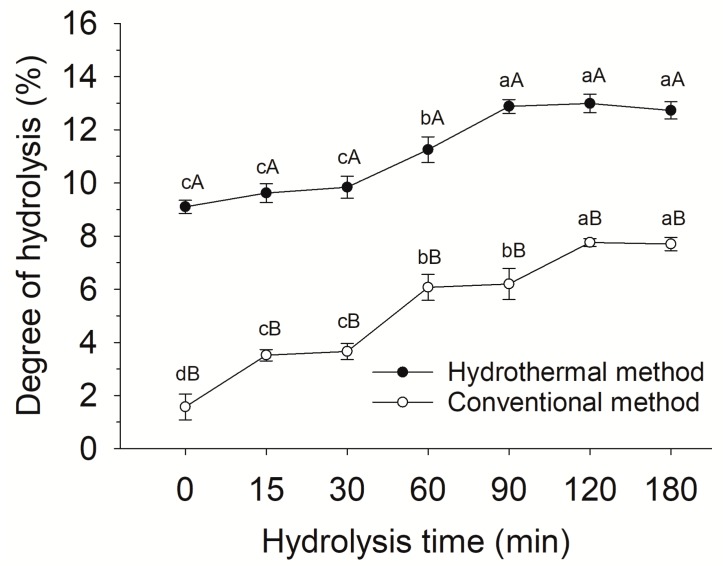
Degree of hydrolysis of gelatin hydrolysates of fish scales treated by hydrothermal and conventional methods followed by alcalase hydrolysis. Different small letters indicate that the results at different hydrolysis time within the sample differ significantly (*p* < 0.05). Different capital letters indicate that the results between the samples treated by two methods differ significantly (*p* < 0.05).

The conventionally treated fish scales showed almost no DH before enzyme hydrolysis. Interestingly, the hydrothermal treated samples displayed a DH of 9.1% at 0 min (Figure 3), indicating that some protein molecules had been cleaved into peptides or amino acids during the hydrothermal process. Under the hydrothermal condition, water acquires unique properties, such as increased ionic products and enhanced hydrogen bonding interactions [10], which could cause partial hydrolysis of proteins into smaller molecules. The effects of hydrothermal pretreatment on the hydrolysis efficiency of fish scales was studied over a period of 180 min (Figure 3). There was a significant (*p* < 0.05) effect of hydrolysis time on the DH of hydrolysates. The DH of the fish scales treated by conventional method increased rapidly from 1.6% to 7.8% with the increasing hydrolysis time in the period of 180 min. The DH of the hydrothermal treated samples increased gradually from 9.1% to 12.9% at the first 90 min of enzymatic hydrolysis, after which there was no obvious change. In addition, the DH of the hydrolysates treated by hydrothermal process was significantly (*p* < 0.05) higher than that of the conventional treated samples. High temperature during the hydrothermal process might unfold protein molecules and expose inner sites, so that commercial enzymes could attack them more easily [33]. Similar changes are also found in fish skin [11] and bone [14].

**Figure 4:**
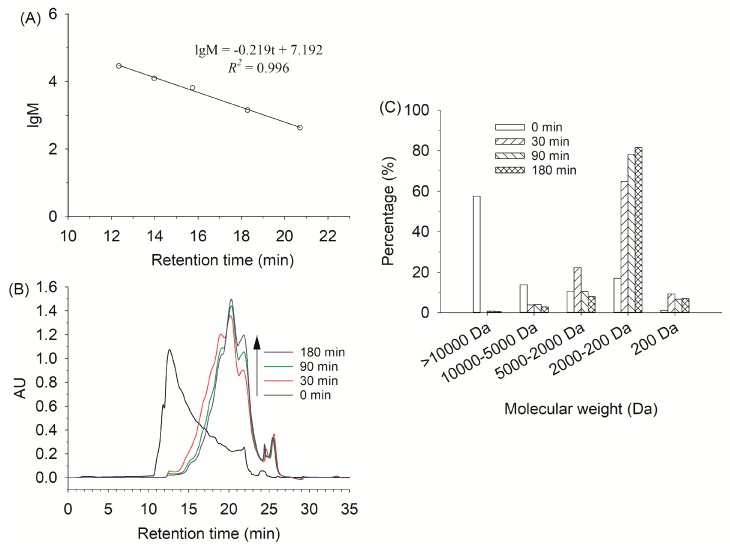
A standard curve (**A**) of molecular weight standards and their retention time using TSK-Gel G2000 SWxl column for analysis. Standards: carbonic anhydrase (29,000 Da), cytochrome C (12,500 Da), aprotinin (6,511.4 Da), bacitracin (1,422.69 Da) and hippuryl-histidyl-leucine (429 Da). Gel filtration chromatogram (**B**) and molecular weight distribution (**C**) of gelatin hydrolysates for different hydrolysis times (0–180 min).

Molecular weight distributions of the hydrolysates were analyzed by gel filtration chromatography. As shown in Figure 4, about 13.72% and 27.55% of the total hydrolysates ranged in the 5000–10000 Da and 200–5000 Da at 0 min. The majority of protein were leached from fish scales at 135 °C, accompanied by the depolymerization of collagen into gelatin and peptides. The contents of peptides less than 2000 Da increased when alcalase was added to the reaction solution. In particular, peptide contents increased obviously from 16.96% to 78.12% at 90 min, ranging from 200 to 2000 Da. These data were closely consistent to the DH curves, indicating that proteins would degrade into polypeptides and small peptides as hydrolysis progressed. Accordingly, gelatin hydrolysates show potent biological activities that are closely related to the presence of small peptides [1]. When the hydrolysis time was extended to 180 min, no obvious change was found in molecular weight distribution of the gelatin hydrolysates. 

### 2.4. Amino Acid Composition Analysis

The total amino acid content in tilapia scale, residue, tilapia scale protein powder, and gelatin hydrolysates were 51.68%, 14.31%, 91.13% and 92.63%, respectively. The amino acid composition of tilapia scale samples per 1000 total residues was shown in Table 1. Among all the samples, glycine was the most abundant amino acid, making up around 315.2–372.7 residues per 1000 residues. After hydrothermal treatment, the amino acid content of tilapia scale protein powder increased significantly (*p* < 0.05) compared with that of untreated fish scales. It had high levels of proline, alanine, glutamic acid, hydroxyproline and arginine, but tiny amounts of tyrosine, histidine and methionine. The amino acid profiles of the extracted protein were quite similar to those of collagen/gelatin extracted by extrusion-hydro-extraction process [19]. Especially, the amino acid (proline and hydroxyproline) content of the scale protein powder was 208.2 residues per 1000 total residues, which was similar to that of tilapia (*Tilapia zillii*) scale gelatin (212) [34], and higher than that of tilapia (*Oreochromis* sp.) scale collagen extracted by an extrusion–hydro-extraction process (187.7) [19]. In the tilapia scale protein powder, the content of histidine, threonine, methionine, valine, phenylalanine + tyrosine, isoleucine, leucine, and lysine was 0.89 g/100 g, 2.49 g/100 g, 1.14 g/100 g, 2.36 g/100 g, 2.52 g/100 g, 1.41 g/100 g, 2.79g/100 g and 3.53 g/100 g, respectively. All the essential amino acid content was 18.80% in the total amino acids. In addition, although most amino acid residues were high in the tilapia scale residue sample, its amino acid content was only 14.31%, indicating that most of the protein was recovered through hydrothermal process.

In spite of the deficiency in some essential amino acids, the recovered scale protein and gelatin hydrolysates might be used to improve water holding capacity of protein gel [35] or as a natural clarifier for plant beverages [36]. Many studies have indicated a good correlation between the bioactivity of protein hydrolysates and certain amino acid residues [37]. Accordingly, food-borne peptides with high levels of glycine, proline, glutamic acid and alanine, would possess strong ACE inhibitory activity [38]. Therefore, the gelatin hydrolysate produced by hydrothermal treatment followed by alcalase hydrolysis might be expected to present optimal ACE inhibitory ability because of the high level of these amino acid residues.

### 2.5. ACE Inhibitory Activity of Gelatin Hydrolysates

**Figure 5:**
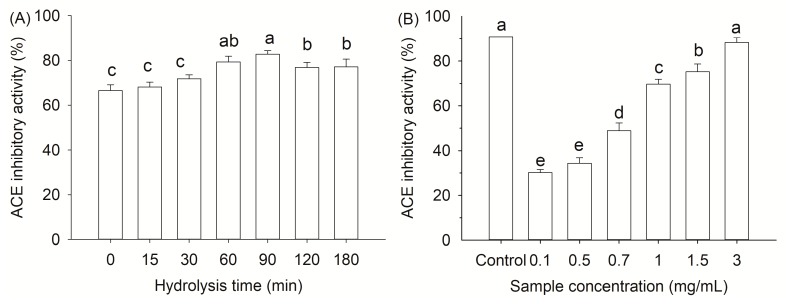
Effect of hydrolysis times (**A**) and sample concentration (**B**) on ACE inhibitory activity of gelatin hydrolysates. Captopril (10 μM) served as a positive control. Different letters indicate that the results differ significantly (*p* < 0.05).

ACE is known to hydrolyze angiotensin I into angiotensin II and degrade bradykinin, causing high blood pressure. ACE inhibitors could prevent the generation of angiotensin II, thus protecting against cardiovascular diseases. Up to now, food-derived peptides have been extensively studied to exhibit ACE inhibitory activity, which are generally considered safer and milder than synthetic drugs, such as captopril and enalapril [37,39]. In this sense, ACE inhibitory activity of the gelatin hydrolysates was analyzed. The hydrothermal treated samples showed a high ACE inhibitory activity of 66.62% before enzymatic hydrolysis (Figure 5A). It suggested that some peptides with ACE inhibitory ability were released under hydrothermal condition even without enzymatic hydrolysis. The ACE inhibitory activity increased with hydrolysis time and reached a maximum after 90 min of hydrolysis. The result was consistent with the results of degree of hydrolysis (Figure 3). However, the ACE inhibition activity decreased significantly (*p* < 0.05) at longer hydrolysis times, suggesting further hydrolysis of the active peptides or a formation of less inhibitory peptides [40]. The results agreed with the work of Barzideh et al. in which they found that peptide size was not the only important role in defining its bioactivity [41]. Actually, the peptide size, degrees of hydrolysis and amino acid sequence of gelatin hydrolysates are all important in determining the bioactivity.

ACE inhibitory activity of the gelatin hydrolysates changed as a function of protein concentration (0.1–3.0 mg/mL), which ranged from 30.2% to 88.3% in a protein concentration dependent fashion (Figure 5B). The IC_50_ value of the gelatin hydrolysate was 0.73 mg/mL. Compared to the captopril (IC_50_ = 1.7 ng/mL) [42], the gelatin hydrolysate showed a milder inhibitory activity against ACE. The activity was lower than that of chicken collagen hydrolysate by an *Aspergillus* species-derived enzyme (IC_50_ = 0.26 mg/mL) [43], but higher than that of gelatin hydrolysate obtained from grass carp fish (*Ctenopharyngodon idella*) scale (IC_50_ = 1.66 mg/mL) without further purification [44]. Chen et al. also evaluated the ACE inhibitory ability of acid soluble collagen hydrolysates from lizardfish (*Synodus fuscus*) scale after hydrolysis by different commercial proteinases and obtained IC_50_ values between 0.12–2.14 mg/mL [45]. The present results suggest that hydrothermal process is an effective pretreatment for the release of peptides with ACE inhibitory activity from fish scale during hydrolysis. However, further identification of peptide sequences and in vivo experimentation are necessary.

### 2.6. Stability of ACE Inhibitory Activity

**Figure 6:**
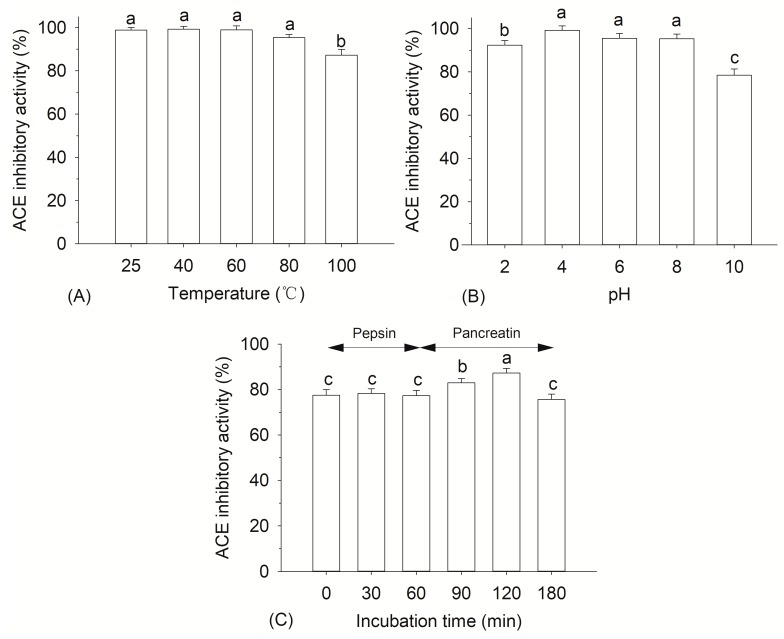
Thermal (**A**), pH (**B**) and digestive (**C**) stabilities of gelatin hydrolysates with the highest DH. Different letters indicate that the results differ significantly (*p* < 0.05).

The bioactivity of protein hydrolysates might be affected by processing conditions, such as pH, heat and high pressure, when applied in food systems [46]. As shown in Figure 6A, the gelatin hydrolysates retained ACE inhibitory activity after various heat treatments, except for 100 °C. The activity loss was only about 13% after heating at 100 °C for 120 min. In this study, the gelatin hydrolysates showed good tolerance at pH 4–8 (Figure 6B). In strong alkaline (pH 10.0) or acid (pH 2.0) conditions, there was a slight reduction of ACE inhibitory activity. It could be caused by the partial degradation of peptides into inactive fragments under these conditions.

In vitro simulated gastrointestinal digestion helps in understanding the digestive behavior of peptides in gelatin hydrolysates after oral administration [28]. As shown in Figure 6C, after pepsin digestion, the gelatin hydrolysates maintained strong ACE inhibitory activity. With further hydrolysis during simulated intestinal digestion, there was a significant increase in ACE inhibitory activity with 120 min (*p* < 0.05) but decreased at the end of digestion. After gastrointestinal digestion, some new peptides with a lower molecule weight were probably produced, which might have higher ACE inhibitory activity [47]. A previous study reported that no change in the ACE inhibitory activity of Pacific hake (*Merluccius productus*) protein hydrolysate during in vitro simulated gastrointestinal digestion, however, a purified peptide fraction presented decreased or no ACE inhibitory activity after digestion [48]. In the present work, the results indicated that the gelatin hydrolysates were stable against digestive enzymes degradation within the gastrointestinal tract [49]. 

## 3. Materials and Methods 

### 3.1. Materials

Fresh tilapia (*Oreochromis niloticus*) scales were provided by Zhanjiang Universal Seafood Corp. (Zhanjiang, Guangdong, China). The scales were washed with tap water, packed in plastic bags, frozen at the factory and then transported on ice to the lab within 2 days. Once they arrived, the scales were immediately stored at −18 °C until use. Alcalase 3.0T was purchased from the Chinese branch of the Danish company Novozyme (Tianjin, China). Angiotensin I-converting enzyme (ACE) from rabbit lungs, hippuric acid (HA), Hippuryl-histidyl-leucine (HHL), pepsin, pancreatin, L-isoleucine, bacitracin (1422.69 Da), aprotinin (6511.4 Da), cytochrome C (12,500 Da), carbonic anhydrase (29,000 Da), *o*-pthaldialdehyde (OPA) and captopril were obtained from Sigma-Aldrich (Milwaukee, WI, USA). All other reagents with an analytical grade were purchased from Sinopharm Chemical Reagent Co., Ltd. (Shanghai, China). 

### 3.2. Fish Scales Pretreatment

Frozen fish scales were mixed with distilled water with a ratio of 2–12% and then hydrolyzed hydrothermally at 85–135 °C for 15–120 min. The insoluble fish scale residues were removed by centrifugation at 8,000 g for 10 min and the supernatants were collected. After being freeze-dried with a lyophilizer (Freezone 2.5L, LABCONCO, Kansas City, MO, USA), tilapia scale protein powder was obtained and stored at −18 °C. Frozen fish scales were also treated by the conventional method as described by Wang et al. [23] with some modification. The scales were immersed in 0.1 M HCl for 2 h for decalcification and then washed with tap water until neutral to obtain the decalcified scales. The decalcified scales were mixed with distilled water (1:20, *w*/*v*) and heated at 60 °C for 2 h. The gelatin solution was obtained after centrifugation at 8000× *g* for 10 min. In addition, frozen fish scales were also dried at 50 °C for 12 h and then ground with an IKA A11 basic analytical mill (IKA, Staufen, Germany) to get the ground scales. As a comparison, the obtained ground scales and decalcified scales were further treated at 135 °C for 90 min to verify whether decalcification or pulverization is necessary for fish scales during hydrothermal treatment.

### 3.3. Preparation of Tilapia Scale Protein Hydrolysates

Twenty grams of tilapia scale protein powder were resuspended in 1 L distilled water. The powder was hydrolyzed by alcalase with an enzyme-to-substrate ratio (E:S) of 1:100 (*w*/*w*) at the condition of 55 °C, pH 8.0 and 125 rpm in a rotary thermostatic oscillator. During the hydrolysis reaction, the pH was maintained by periodic addition of 0.1 M NaOH solution. The hydrolysate was taken periodically at the appropriate time and heated immediately at 100 °C for 10 min to inactivate the protease activity. After cooling, the solution was centrifuged at 8000× *g* for 10 min. The supernatant was collected and frozen at −18 °C for further analysis. For comparison, the gelatin solution obtained by the conventional method was hydrolyzed at the same condition.

### 3.4. Chemical Analysis of Tilapia Scale Protein Powder

The determination of the moisture, fat, ash and crude protein were performed using the Association of Official Analytical Chemists (AOAC) methods [50]. Crude protein (N × 5.95) was measured by the Kjeldahl method. The moisture content was measured by oven drying at 105 °C for 8 h. Fat analysis was carried out using the Soxhlet extraction method. Ash content was determined by incineration at 580 °C for 8 h. 

### 3.5. Determination of Protein Recovery

The soluble nitrogen in the supernatant and total nitrogen in fish scale were determined by the Kjeldahl method [50]. The protein recovery of fish scale was expressed as the soluble nitrogen in the supernatant divided by the total nitrogen in fish scale.

### 3.6. Amino Acid Composition Analysis

For the total amino acid analysis, the samples were hydrolyzed in 6 M HCl under vacuum at 110 °C for 22 h in sealed tubes. The determination of amino acids in the sample was made on an Agilent 1100 series HPLC system (Agilent Technologies, Palo Alto, CA, USA) equipped with an ODS Hypersil (4.6 mm × 250 mm) column. The detection wavelength was 338 nm, except for hydroxyproline, which was detected at 262 nm.

### 3.7. Fourier Transform Infrared (FTIR) Spectroscopy

FTIR spectra of fish scale were obtained by a FTIR spectrometer (Bruker Optics GmBH, Ettlingen, Germany). The assay was performed using the potassium bromide pellet sampling technique according to the method of Elavarasan et al. [28]. All sample spectra were obtained from 4000 to 400 cm^−1^ and recorded at 128 scans and 4 cm^−1^ resolution.

### 3.8. Determination of the Degree of Hydrolysis

The degree of hydrolysis (DH) is defined as the percentage of free amino nitrogen cleaved from protein, which was expressed as the ratio of amino-nitrogen to total nitrogen of the sample [14]. The amount of α-amino nitrogen was quantified with the *o*-phthaldialdehyde (OPA) method according to Nielsen, et al. [51] with some modifications. The determination was carried out by the addition of 400 μL of the samples to the OPA reagent (3 mL). The absorbance of the mixture was then measured at 340 nm with an UV spectrophotometer (Thermo Fisher Scientific, Inc., Waltham, MA, USA) after 2 min. The free amino group content was expressed in terms of l-isoleucine. 

### 3.9. Molecular Weight distribution

The molecular weight distribution of the hydrolysates was characterized by a Waters 2695 HPLC system fitted with a TSK-Gel G2000 SW_XL_ column (7.8 mm × 300 mm, Tosoh, Tokyo, Japan) and a TSK-Gel SW_XL_ guard column (6.0 mm × 40 mm, Tosoh, Tokyo, Japan). The supernatant (20 μL) was loaded onto the column after filtering with a 0.45 μm membrane filter. The elution was carried out using 45% (*v*/*v*) acetonitrile containing 0.1% (*v*/*v*) trifluoroacetic acid (TFA) at 0.5 mL/min and its absorbance was monitored at 220 nm. A molecular weight calibration curve (lg(M) = −0.219t + 7.192, *R*^2^ = 0.996) was obtained using a set of standards from Sigma-Aldrich: HHL (429 Da), bacitracin (1,422.69 Da), aprotinin (6,511.4 Da), cytochrome C (12,500 Da) and carbonic anhydrase (29,000 Da). 

### 3.10. Determination of ACE Inhibitory Activity

The ACE inhibitory activity was determined by the method of Wu et al. [52] with some modifications. Briefly, 40 μL sample was mixed with 25 μL ACE solution (100 U/L) in a 2.0 mL polyethylene centrifuge tube. After incubation in a 37 °C water bath for 10 min, 40 μL of the substrate (6.5 mM HHL in 0.1 M borate buffer, containing 0.3 M NaCl, pH 8.3) was added into the mixture to initiate the reaction. The reaction was terminated by the addition of 85 μL of 1 M HCl after incubation for 30 min. After filtration through a 0.22 μm membrane filter, hippuric acid (HA) was separated by a Waters e2695 HPLC system (Waters Corp., Milford, MA, USA) equipped with a SunFire C18 column (4.6 mm × 250 mm, 5 μm). The elution was carried out with 30% (*v*/*v*) acetonitrile containing 0.1% (*v*/*v*) TFA at 0.8 mL/min and its absorbance was monitored at 228 nm. IC50 value was defined as the concentration of ACE inhibitors needed to reduce activity by 50%. A saline solution was used as a negative control, and captopril served as a positive control. ACE-inhibitory activity (%) was calculated as follows:

ACE inhibitory activity (%) = (A − B)/A × 100(1)

where A is the HA content of the control (without the sample) and B is the HA content of the reaction with the sample.

### 3.11. Stability of ACE Inhibitory Activity

The thermal and pH stability was measured according to the method of Ketnawa et al. [46]. The gelatin hydrolysates (10 mg/mL) were incubated at various temperatures 20, 40, 60, 80 and 100 °C for 2 h, respectively. The hydrolysates were also adjusted to pH 2, 4, 6, 8 and 10 with 1 M HCl or NaOH and incubated at 37 °C for 2 h. All samples were cooled to room temperature and pH of the solutions was then adjusted to 8.3. The residual ACE inhibitory activity was determined as described above.

In vitro gastrointestinal digestion was performed according to Ketnawa et al. [46] with some modification. Gelatin hydrolysate solution (10 mg/mL; 50 mL) was adjusted to pH 2.0 using 1 M HCl, incubated at 37 °C for 2 min, and then mixed with 20 mg pepsin in 1 ml of 0.1 M HCl. The mixture was gently shaken at 37 °C for 60 min. The pH of the reaction solution was raised to 7.5 using 1 M NaOH. The solution was then mixed with 50 mg pancreatin and incubated for an additional 120 min. The samples were randomly taken at 0, 30, 60, 90, 120 and 180 min during the in vitro digestion and quickly placed in a boiling water bath for 10 min followed by centrifugation with the supernatant for ACE inhibitory activity determination.

### 3.12. Statistical Analysis

The data were expressed as mean ± standard deviation (Mean ± SD) and analyzed by variance analysis (ANOVA) using software SPSS 17.0 (SPSS Inc., Chicago, IL, USA). Significant difference between means were identified using Duncan’s Multiple Ranger Test (*p* < 0.05). 

## 4. Conclusions

Hydrothermal treatment was confirmed to be a good method for the recovery of tilapia scale protein. An extraction rate of 84.81% was obtained from fish scales treated at 135 °C for 90 min. The hydrothermal pretreatment enhanced protein recovery and the degree of hydrolysis of fish scales without demineralization. The obtained gelatin hydrolysates mainly distributed in the range of 200–2000 Da with an ACE IC_50_ value of 0.73 mg/mL after 90 min of hydrolysis by alcalase. The ACE inhibitory activity of gelatin hydrolysates was stable under high temperature, pH and gastrointestinal proteases. Further studies on the identification of bioactive peptides, in vivo experiments and the functionality of scale gelatin hydrolysates in food-related process are currently underway. 

## Figures and Tables

**Table 1:** Amino acids composition of tilapia scale, residue, tilapia scale protein powder, and gelatin hydrolysates (results are expressed as residues/1000 total residues).

Amino Acids	Tilapia Scale	Residue	Tilapia Scale Protein Powder	Gelatin Hydrolysate
Aspartic acid/asparagine	50.8 ± 0.6 b	60.9 ± 0.5 a	47.6 ± 0.6 c	47.9 ± 1.1 c
Glutamic acid/glutamine	84.5 ± 0.5 b	102.9 ± 0.7 a	81.4 ± 0.4 b	82.3 ± 0.6 b
Serine	28.7 ± 0.7 b	37.9 ± 0.4 a	29.8 ± 0.7 b	29.5 ± 0.5 b
Histidine	9.4 ± 0.1 b	22.6 ± 0.4 a	6.7 ± 0.2 c	8.8 ± 0.7 b
Glycine	358.3 ± 0.3 b	315.2 ± 0.9 c	372.7 ± 1.7 a	362.9 ± 1.4 b
Threonine	27.0 ± 0.5 b	37.4 ± 0.7 a	24.4 ± 0.8 b	25.8 ± 0.3 b
Arginine	53.0 ± 0.5 a	48.0 ± 0.4 b	53.6 ± 0.4 a	51.6 ± 1.1 a
Alanine	125.0 ± 0.8 a	101.7 ± 0.6 b	129.1 ± 0.9 a	124.6 ± 0.6 a
Tyrosine	4.4 ± 0.1 b	12.5 ± 0.2 a	2.6 ± 1.4 b	4.3 ± 0.4 b
Valine	27.1 ± 0.2 b	46.7 ± 0.5 a	23.5 ± 0.6 b	26.4 ± 0.7 b
Methionine	7.4 ± 0.4 a	0.1 ± 0.0 b	8.9 ± 0.3 a	10.3 ± 0.4 a
Phenylalanine	15.9 ± 0.3 b	21.3 ± 0.4 a	14.9 ± 0.5 b	15.5 ± 0.7 b
Isoleucine	14.7 ± 0.2 b	26.2 ± 0.5 a	12.5 ± 0.6 b	14.3 ± 0.5 b
Leucine	27.4 ± 0.4 b	41.7 ± 0.6 a	24.8 ± 0.7 b	26.5 ± 0.9 b
Lysine	26.4 ±0.7 a	23.5 ± 0.2 b	28.2 ± 0.7 a	27.3 ± 0.5 a
Proline	140.1 ± 0.3 a	101.7 ± 0.6 b	139.3 ± 0.9 a	142.1 ± 1.1 a
Hydroxyproline	71.5 ± 0.8 a	38.1 ± 0.9 b	68.9 ± 1.3 a	70.1 ± 0.9 a
Total	1000	1000	1000	1000

Values with different letters within the same row differ significantly (*p* < 0.05).
